# Bu Shen Yi Sui Capsule Promotes Myelin Repair by Modulating the Transformation of A1/A2 Reactive Astrocytes *In Vivo* and *In Vitro*

**DOI:** 10.1155/2022/3800004

**Published:** 2022-09-01

**Authors:** Zheng Zha, Yi-Jiang Liu, Si-Si Liu, Nan Zhang, Jun-Ling Li, Fang Qi, Liang-Yun Jin, Bing Xue, Tao Yang, Yong-Ping Fan, Hui Zhao, Lei Wang

**Affiliations:** ^1^School of Traditional Chinese Medicine, Beijing Key Lab of TCM Collateral Disease Theory Research, Capital Medical University, Beijing 100069, China; ^2^Core Facility Center, Capital Medical University, Beijing 100069, China; ^3^Beijing Tian Tan Hospital, Capital Medical University, Beijing 100070, China

## Abstract

*Background/Aims*. Multiple sclerosis (MS) is an autoimmune disorder that affects the central nervous system (CNS) primarily hallmarked by neuroinflammation and demyelination. The activation of astrocytes exerts double-edged sword effects, which perform an integral function in demyelination and remyelination. In this research, we examined the therapeutic effects of the Bu Shen Yi Sui capsule (BSYS), a traditional Chinese medicine prescription, in a cuprizone- (CPZ-) triggered demyelination model of MS (CPZ mice). This research intended to evaluate if BSYS might promote remyelination by shifting A1 astrocytes to A2 astrocytes. *Methods*. The effects of BSYS on astrocyte polarization and the potential mechanisms were explored *in vitro* and *in vivo* utilizing real-time quantitative reverse transcription PCR, immunofluorescence, and Western blotting. Histopathology, expression of inflammatory cytokines (IL-10, IL-1*β*, and IL-6), growth factors (TGF-*β*, BDNF), and motor coordination were assessed to verify the effects of BSYS (3.02 g/kg/d) on CPZ mice. In vitro, A1 astrocytes were induced by TNF-*α* (30 ng/mL), IL-1*α* (3 ng/mL), and C1q (400 ng/mL), following which the effect of BSYS-containing serum (concentration of 15%) on the transformation of A1/A2 reactive astrocytes was also evaluated. *Results and Conclusions*. BSYS treatment improved motor function in CPZ mice as assessed by rotarod tests. Intragastric administration of BSYS considerably lowered the proportion of A1 astrocytes, but the number of A2 astrocytes, MOG+, PLP+, CNPase+, and MBP+ cells was upregulated. Meanwhile, dysregulation of glutathione peroxidase, malondialdehyde, and superoxide dismutase was reversed in CPZ mice after treatment with BSYS. In addition, the lesion area and expression of proinflammatory cytokines were decreased and neuronal protection factors and anti-inflammatory cytokines were increased. *In vitro*, BSYS-containing serum suppressed the A1 astrocytic markers' expression and elevated the expression levels of A2 markers in primary astrocytes triggered by C1q, TNF-*α*, and IL-1*α*. Importantly, the miR-155/SOCS1 signaling pathway was involved in the modulation of the A1/A2 phenotype shift. Overall, this study demonstrated that BSYS has neuroprotective effects in myelin repair by modulating astrocyte polarization via the miR-155/SOCS1 pathway.

## 1. Introduction

Multiple sclerosis (MS) is a heterogeneous neurodegenerative condition [1] that leads to nontraumatic neurological disability as a result of demyelination and axonal injury of the central nervous system (CNS) [2], for which there is still a lack of effective therapies. Approximately 2.8 million people are estimated to have MS globally [3], which imposes a heavy burden on individuals and their families. Accumulating evidence shows that autoimmune-mediated neuroinflammation is associated with inefficient remyelination [4], leading to the deteriorated repair of demyelinating lesions, which might influence the poor prognosis of MS [5].

The activation of astrocytes and microglia were upregulated dramatically within the demyelination foci, implying that neuroinflammation initiated by innate immune resident cells of the CNS may result in axonal injury and oligodendrocyte loss [6]. Astrocytes, which are the most predominant glial cells found in the brain, perform crucial functions in homeostasis and response to CNS injury [7]. Astrocyte polarization has been widely studied in recent years, and the astrocytic phenotype has been well elucidated. Astrocytes play a harmful or favorable role depending on their polarization phenotypes. According to the findings of a series of research reports, reactive astrocytes may be classified into two main categories: the A1 phenotype has neurotoxic characteristics, whereas the A2 astrocytes are considered to be neurotrophic and can provide neuroprotection [8]. In MS, astrocytes transform to the A1 phenotype [9], secreting several neurotoxic cytokines, such as tumor necrosis factor-alpha (TNF-*α*), interleukin-1*β* (IL-1*β*), and IL-6, leading to the formation of a proinflammatory milieu in the CNS and the subsequent myelin injury as well as impaired remyelination process [10]. Therefore, the development of new drugs that skew A2 polarization to increase the production of anti-inflammatory cytokines and neuroprotective mediators and thus enhance myelin repair may provide an effective therapeutic strategy for MS [11].

MicroRNAs (miRs) are small noncoding RNAs, containing approximately 21 nucleotides, that can downregulate messenger RNA (mRNA) expression by targeting the three prime untranslated regions (3′UTR) [12]. In recent years, several research reports have indicated that specific miRs play an instrumental function in the process of demyelination and remyelination [13], which are critical in MS pathologies [14]. Particularly, upregulation of miR-155 was shown to inhibit the repair of myelin by promoting a proinflammatory microenvironment in the CNS [15, 16]. Moreover, miR-155 was confirmed to drive the polarization of astrocytes towards the neurotoxic phenotype (A1) via regulating the suppressor of the cytokine signaling 1 (SOCS1) pathway [17]. Several human neurodegenerative conditions, including MS, have been linked to upregulated expression levels of A1 astrocytes. The A1 phenotype induces oligodendrocytes death and inhibits oligodendrocyte precursor cells (OPCs) differentiation and maturation, thereby exacerbating demyelination and preventing myelin repair [9]. In MS, oligodendrocytes are the target of inflammatory and immune attacks and their progressive death leads to demyelinated lesions and remyelination failure [18]. The A2-like type enhances the maturity of OPCs and protects against white matter injury progression. Consequently, because miR-155 regulates the transition of A1/A2 reactive astrocytes, we hypothesized that reducing its expression level might be a viable treatment strategy for myelin repair in MS.

Growing evidence has illustrated that traditional therapies are successful in the treatment of neurodegenerative diseases (ND) of the CNS. Traditional therapies in China and India provide a holistic treatment for ND. The herbs and formulations of Ayurveda, traditional Chinese medicine (TCM), are rich in antioxidants, immunoregulatory, neuroprotective, and anti-inflammatory compounds, which have been shown to regulate neuroendocrine-immune functions, restore brain function, and improve quality of life [19]. The works of Sharma et al. have well summarized the therapeutic roles of Ayurveda, TCM, and other alternative therapies in epilepsy, depression, Parkinson's disease (PD), and Alzheimer's disease (AD). It lays a solid theoretical foundation for the development of novel drugs in ND and paves the pathways for further study on traditional herbal medicine as well as for its standardization in the future [20]. In recent years, Chinese herbal medicine has made significant contributions to the prevention and treatment of MS [21]. It has been effective in modulating immune response [22], reducing neuroinflammation, alleviating nerve injury [23], and strengthening myelin repair [24]. For more than a decade, the Bu Shen Yi Sui capsule (BSYS), a traditional Chinese medicine, has been utilized in clinical trials to treat MS. This formulation consists of several Chinese medicines including *Rehmanniae Radix* (Shengdihuang), *Rehmanniae Radix Praeparata* (Shudihuang), *Polygoni Multiflori Radix Praeparata* (Heshouwu), *Forsythiae Fructus* (Lianqiao), *Gastrodiae Rhiazoma* (Tianma)*, Scorpio* (Quanxie), *Hirudo* (Shuizhi)*, Fritillariae Thunbergii Bulbus* (Zhebeimu), *Rhei Radix et Rhizoma* (Dahuang), and *Leonuri Herba* (Yimucao). Our earlier research illustrated that the therapeutic impacts of BSYS in experimental autoimmune encephalomyelitis (EAE) mice were linked to the promotion of microglia polarization towards M2, which could be associated with alterations in miR-155 and miR-124 *in vivo* [25]. Nevertheless, it remains unclear whether BSYS can directly modulate astrocyte polarization in a cuprizone- (CPZ-) triggered demyelination model of MS (CPZ mice). Given BSYS's antineuroinflammatory effects, it would be quite interesting to examine whether it could be utilized to enhance myelin repair by altering astrocyte polarization, which would be a significant advancement. The focus of this research was to investigate the proremyelinating impacts of BSYS on CPZ mice, as well as the possible processes of astrocytic polarization modulation by BSYS.

## 2. Materials and Methods

### 2.1. Animals and Drugs

All animal experimentations were done in compliance with the standards stipulated in the guidelines of the International Council for Laboratory Animal Science, and the approval was granted by the Capital Medical University's Animal Experiments and Experimental Animal Welfare Committee (permit number: AEEI-2020-181). Female C57BL/6 J mice between the ages of 6 and 8 weeks and weighed between 15 and 18 g were procured from Huafukang Biotechnology Co., Ltd. (Beijing, China). We housed the mice in a specific pathogen-free (SPF) laboratory at the Capital Medical University's Experimental Animal Center (SYXK (Beijing) 2018-0003). The animals were given water and food *ad libitum* under constant humidity (40-50%) and temperature (22 ± 3°C), as well as a 12-hour darkness/light cycle. Regular rodent chow that contained 0.2% CPZ (Sigma-Aldrich, United States) was provided by Keao Xieli Feed Co., Ltd. (Beijing, China). Asia-East Biopharmaceutical Co., Ltd. (Beijing, China) synthesized and supplied the BSYS utilized in this experiment. The production process of BSYS was similar to that in our previous reports [26]. BSYS was composed of *Rehmanniae Radix Praeparata*, *Rehmanniae Radix*, *Polygoni Multiflori Radix Praeparata*, *Rhei Radix et Rhizoma*, *Fritillariae Thunbergii Bulbus*, *Hirudo, Scorpio*, *Gastrodiae Rhiazoma, Forsythiae Fructus*, and *Leonuri Herba*. These herbs were combined in the following ratios: 10 : 10 : 10 : 2 : 6 : 3 : 2 : 3 : 6 : 10. All herbs, except *Fritillariae Thunbergii Bulbus*, were immersed for half an hour in water that had been distilled and then subjected to heating for two hours until boiling. After the inspissation of the filtered solution was carried out at a lowered pressure and a temperature of 70°C, the dry powdered sample was mixed evenly with the flour that was made from *Fritillariae Thunbergii Bulbus*. The powder mixture that was produced was then put into capsules. The chemical components of BSYS were identified and quantified by UPLC-QTOF-MS/MS and UPLC-LTQ-Orbitrap-MSn, which have been published elsewhere [27].

### 2.2. Establishment of Demyelination Model and Drug Treatment

**Figure 1:**
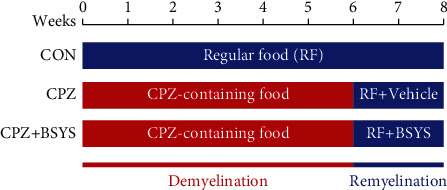
Schematic timeline of the experimental procedures. Blue represents regular food (RF), whereas red represents a diet containing 0.2% CPZ (CPZ-containing food). In the CPZ mouse model, the first 6 weeks are the demyelination phase and the last 2 weeks are the remyelination phase.

The experimental mice were categorized at random into three groups: the normal control group (CON), CPZ model group (CPZ), and BSYS treatment group (CPZ+BSYS). In a paper published by our team in 2018, Zhao et al. tested the drug-only group by gavage of CON mice with BSYS 3.02 g/kg/d. They found no significant difference between mice in the CON group and mice in the CON+BSYS group [26]. Therefore, the BSYS drug-only control group was not retained in this experiment. Dietary supplements of CPZ were not supplied to the animals in the CON group. The CPZ-mediated demyelination model was established as previously described; to trigger demyelination, the mice received a diet that contains 0.2 percent (0.2% *w*/*w*) cuprizone for six weeks. Afterward, they were switched to a regular diet for another two weeks. During the recovery period, the mice participants in the BSYS treatment group were subjected to BSYS by gavage at a dose of 3.02 g/kg one time per day for two weeks. In addition, the therapeutic equivalent daily dosage of BSYS administered to the mice was set to be 3.02 g/kg, which was the optimum dose for treating the MS mouse model in our previous study [25, 28], whereas mice in the CPZ group were administered an equivalent volume of vehicle (distilled water) (Figure 1).

### 2.3. Body Weight Measurement and the Rotarod Test

After receiving CPZ-containing chow, each mouse's weight was recorded two times a week starting on the initial day of treatment. Subsequently, the mice were trained on the rotating rod for 180 s per day for three days before the behavioral assessment. A rotarod test was administered twice weekly to the mice once they had become acclimated to the apparatus. For 180 seconds, the rotation speed was steadily raised from 5 rpm to 40 rpm. The test was completed when the mouse either dropped off or was able to hold onto the rod for 2 or even more spins [29]. To get an accurate value of the mice's motor function, we recorded the amount of time they spent on the rod.

### 2.4. Luxol Fast Blue (LFB) Staining

The pentobarbital sodium (60 mg/kg) administered intraperitoneally was utilized to anesthetize the mice before perfusing them transcardially with phosphate-buffered saline (PBS), followed by the administration of PBS comprising 4 percent ice-cold paraformaldehyde (PFA). A 12-hour fixation in 4% PFA solution at 4°C and subsequent dehydration and embedding in paraffin were performed after dissection of the brain tissues. Brain paraffin coronal sections (5 *μ*m thick) were prepared, including the corpus callosum (CC). The extent of remyelination was assessed using LFB staining, and the sections were viewed and digitally photographed using a microscope. Three microscopic fields were randomly sampled from CC, caudoputamen (CPu), and anterior commissure (AC). The LFB results were quantified as the relative expression level of integrated optical density (IOD).

### 2.5. Preparation of BSYS-Containing Serum (BSYS-Serum)

BSYS-serum and blank serum were prepared as previously described [30]. In brief, a dose of BSYS equal to 11.7 g/kg of body weight was administered intragastrically to Sprague-Dawley rats two times daily for one week. On the 7th day of the experiment, blood samples were extracted from the rats that had been sacrificed 2 hours after they had been fed by gavage. The serum was then isolated by centrifuging these samples. To produce blank serum, the equivalent amount of distilled water was administered to the control rats. The main chemical constituents in BSYS-serum analyzed using UPLC-MS/MS have been reported in our previous studies [31].

### 2.6. Cell Culture and Treatment

Primary astrocytes (ProCell, Wuhan, China) were extracted from C57BL/6 mice aged between 1 and 3 days old. The astrocytes were grown in Dulbecco's modified essential medium (DMEM) (Analysis Quiz, Beijing, China) containing 10% fetal bovine serum (FBS; Corning, NewYork, USA) and 1% penicillin-streptomycin (P/S; KeyGen, Nanjing, China) and preserved in 5% CO_2_ at 37°C in a humid incubator. Subsequent tests were carried out using astrocytes of the third generation, which were seeded in six-well plates. Afterward, miR-155 mimics (Lenti-miR-155) and miR-Negative Controls (Lenti-miR-NC) were transfected into astrocytes using a lentivirus system (Hanheng, Shanghai, China) after reaching 30-50% confluence. Once 24 hours had elapsed, the cells were treated with TNF-*α* (30 ng/mL, Novoprotein, Shanghai, China), IL-1*α* (3 ng/mL, Novoprotein, Shanghai, China), and C1q (400 ng/mL, MyBioSource, San Diego, USA), named Astrocyte Stimulation Cocktail (ASC) for 24 h to induce A1 phenotype [9]. Then, BSYS-serum (5%, 10%, 15%, 20%, and 25%) was added to astrocytes and the medium was removed 24 hours later and replaced with a full culture medium. In the next step, the culture medium was obtained after 24 hours and utilized as an astrocyte-conditioned medium (ACM).

BeNa Culture Collection (BNCC, Beijing, China) supplied the OLN-93, an immature oligodendroglial cell line. Afterward, the OLN-93 cells were cultured at 37°C in a 5% CO_2_ humidified atmosphere on 6-well plates in DMEM that contained 10% FBS and 1% P/S. Next, the OLN-93 medium was replaced using DMEM containing 0.5% FBS (low serum differentiation medium (LSM)). The previously collected ACM and LSM were added to a 6-well plate (2.4 mL in total) in the ratio of 1 : 5 (the concentrations of ACM and LSM were 16.7% and 83.3%, respectively) and incubated with OLN-93 for 3 days, to assess the differentiation and the maturation of oligodendrocytes mediated by astrocytes.

### 2.7. Cell Counting Kit-8 (CCK-8) Assay

The CCK-8 test was utilized to ascertain the viability of the cells. Astrocytes (0.8 × 10^4^) were plated in 96-well plates for 24 hours, stimulated by ASC, and subjected to 24 hours treatment with BSYS-serum (5, 10, 15, 20, and 25%). The viability of the cells was evaluated utilizing the CCK-8 kit in a manner consistent with the manufacturer's guidelines. A microplate reader (Molecular Devices, Sunnyvale, USA) was utilized to detect the absorbance at 450 nm in the experiments. The control group's absorbance was interpreted as 100% cell viability.

### 2.8. Immunohistochemical (IHC) and Immunocytofluorescence Staining

The mice's brain tissue segments were deparaffinized and boiled with citrate buffer (pH 6.0) before being blocked in 10% normal goat serum (ZSGB-Bio, Beijing, China) in PBS for 1 hour at 37°C and labeling with rabbit anti-MOG antibody (1 : 500, ab233549, Abcam, Cambridge, UK), rabbit anti-PLP antibody (1 : 400, HA500202, Huabio, Hangzhou, China), rabbit anti-C3 antibody (1 : 200, ab97462, Abcam, Cambridge, UK), rabbit anti-S100A10 antibody (1 : 200, YT4198, Immunoway, Newark, USA), and mouse anti-GFAP antibody (1 : 400, YM3059, Immunoway, Newark, USA) at 4°C throughout the night. Once the sections had been rinsed in PBS, they were incubated at 37°C for 1 hour with Alexa 488-conjugated (1 : 400) and Alexa 594-conjugated (1 : 200) secondary antibodies (ZSGB-Bio, Beijing, China). Antifluorescence quenching (Solarbio, Beijing, China) and 4′,6-diamidino-2-phenylindole (DAPI) mounting medium were employed to label the nuclei.

To perform *in vitro* trials, astrocytes and OLN-93 cells were administrated according to the experimental design. After incubation, cells were fixed for 30 minutes PFA at 4% before being subjected to 5 min of permeabilization using 0.1% Triton X-100 (Beyotime, Shanghai, China) and blocking for 1 h using 5% bovine serum albumin blocking buffer. The next step involved incubating the cells with rabbit anti-C3 antibody (1 : 100, ab97462, Abcam, Cambridge, UK), rabbit anti-S100A10 antibody (1 : 100, PA5-82082, Invitrogen, Carlsbad, USA), mouse anti-GFAP antibody (1 : 200, YM3059, Immunoway, Newark, USA), and anti-PLP antibody (1 : 200, HA500202, Huabio, Hangzhou, China). Once the cells had been rinsed in PBS, they were subjected to 1 hour of incubation at 37°C with CoraLite488-conjugated (1 : 200) and CoraLite594-conjugated-conjugated (1 : 200) secondary antibodies (Proteintech, Wuhan, China) and then counterstained with DAPI for nuclei identification. All pictures were captured with the aid of a fluorescence microscope and analyses were conducted with ImageJ software (National Institutes of Health, Bethesda, USA).

### 2.9. Real-Time Quantitative Reverse Transcription PCR (qRT-PCR)

Total RNA from the CC of the brain tissue and astrocytes was isolated using TRIzol reagent (Thermo Fisher, Waltham, USA) as per the instructions provided by the manufacturer and the concentration measured. qRT-PCR was conducted utilizing the One-Step qRT-PCR kit (Toyobo, Osaka, Japan) with a CFX Connect real-time PCR detection system (Bio-Rad, Hercules, USA) based on the SYBR Green technique. Supplementary Table 1 contains a collection of the primers that were utilized.

### 2.10. Measurement of the Level of GSH-Px, SOD, and MDA

CC homogenate and OLN-93 cell lysates were collected for the determination of malondialdehyde (MDA), superoxide dismutase (SOD), and glutathione peroxidase (GSH-Px). The relative levels of GSH-Px, SOD, and MDA were detected utilizing their corresponding commercial kits (Beyotime, Shanghai, China) as per the guidelines stipulated by the manufacturer.

### 2.11. Western Blot Analysis

From both the CC and cell homogenates, the total protein was isolated. The protein concentration of the cell/tissue lysates was ascertained utilizing the bicinchoninic acid (BCA) protein assay kit (Applygen, Beijing, China). Extracted total proteins were denatured and subjected to electrophoresis separation in sodium dodecyl sulfate-polyacrylamide gel electrophoresis (SDS-PAGE). Total proteins were loaded onto polyvinylidene fluoride (PVDF) membranes (Millipore, Darmstadt, Germany) following the manufacturer's specifications, blocked using StartingBlock blocking buffer (ThermoFisher, Waltham, USA), and subjected to incubation overnight at 4°C in universal antibody diluent (New Cell & Molecular Biotech, Suzhou, China) with primary antibodies against myelin oligodendrocyte glycoprotein (MOG, 1 : 2000, ab233549, Abcam, Cambridge, UK), CNPase (1 : 3000, ab6319, Abcam, Cambridge, UK), myelin basic protein (MBP, 1 : 2500, ab216668, Abcam, Cambridge, UK), proteolipid protein (PLP, 1 : 4000, HA500202, Huabio, Hangzhou, China), C3 (1 : 1000, ab97462, Abcam, Cambridge, UK), S100A10 (1 : 1000, YT4198, Immunoway, Newark, USA), glial fibrillary acidic protein (GFAP, 1 : 3000, YM3059, Immunoway, Newark, USA), IL-1*β* (1 : 1000, 26048-1-AP, Proteintech, Wuhan, China), IL-6 (1 : 1000, # 12912, Cell Signaling Technology, Danvers, USA), IL-10 (1 : 1000, 60269-1-Ig, Proteintech, Wuhan, China), tissue growth factor-*β* (TGF-*β*, 1 : 1000, 19999-1-AP, Proteintech, Wuhan, China), brain-derived neurotrophic factor (BDNF, 1 : 1000, ab108319, Abcam, Cambridge, UK), and *β*-actin (1 : 10000, GTX109639, Genetex, Irvine, USA). The blots were treated for 1 h at an ambient temperature with a horseradish peroxidase-conjugated antibody (Proteintech, Wuhan, China). Immunoblotting bands were identified utilizing enhanced chemiluminescence (ECL, Millipore, Darmstadt, Germany) and visualized on the Fusion FX imaging system (Vilber Lourmat, Torcy, France). When necessary, the membranes were stripped by Western blot stripping buffer (Takara, Kusatsu, Japan) and reprobed with corresponding antibodies. Relative band intensity was quantified by ImageJ software.

### 2.12. Statistical Analysis

GraphPad Prism 7 (GraphPad Software, San Diego, USA) was utilized to undertake all analyses of statistical data. The rotarod data and body weight changes were evaluated utilizing two-way repeated-measures ANOVA with post hoc Turkey tests. Other data from three or more groups were examined with one-way ANOVA accompanied by Turkey's post hoc test. Statistical significance was deemed to have been attained at *P* values of <0.05.

## 3. Results

### 3.1. BSYS Alleviates CPZ-Induced Motor Function Defect

**Figure 2:**
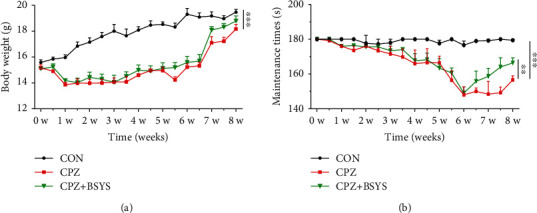
BSYS alleviates CPZ-induced motor function defect. (a) Variations in the mice's body weight per group (*n* = 5). (b) Variations in mouse motor performance as measured by the rotarod test (*n* = 5). The data are presented as mean ± SEM, in comparison to the CPZ group, ^∗∗^*P* < 0.01, ^∗∗∗^*P* < 0.001.

The mice's body weight fluctuations, as well as their locomotor coordination, were measured to examine the therapeutic impacts of BSYS on CPZ mice. CPZ-containing diet significantly decreased the body weight of mice. However, two weeks of BSYS treatment ameliorated CPZ-caused body weight loss, but without statistical significance (Figure 2(a)). CPZ-exposed mice suffered significant motor dysfunction, as shown by a decline in the locomotion time in the rotarod test. Mice subjects that were given CPZ demonstrated decreased latency to fall off a rotarod in contrast with the control group. Additionally, mice that were treated with BSYS had much longer on-rod time in contrast with CPZ mice who were just given a vehicle treatment (Figure 2(b)).

### 3.2. BSYS Enhances Myelin Repair after CPZ-Mediated Demyelination

**Figure 3:**
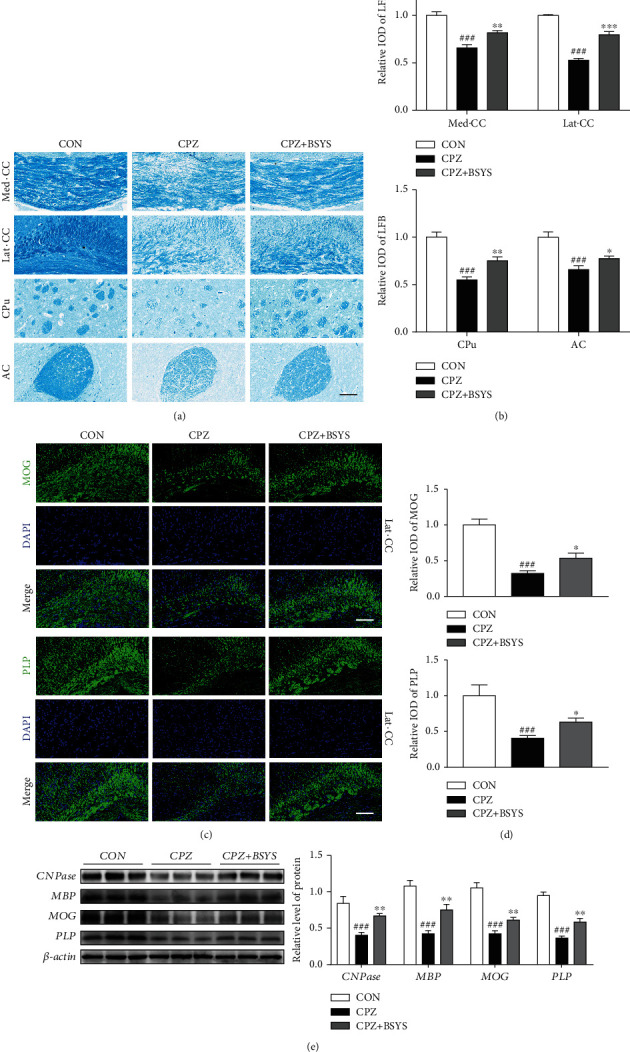
BSYS enhances myelin repair in CPZ-induced demyelination. (a) Changes in demyelination of the Med-CC, Lat-CC, AC, and CPu of mice per group were visualized by LFB staining. Scale bars: 100 *μ*m. (b) Quantification of LFB-stained segments histologically within each group. (c) The MOG and PLP expressions in Lat-CC were detected by immunofluorescence staining. Scale bars: 100 *μ*m. (d) Relative IOD expression of MOG and PLP in Lat-CC. (e) The relative protein expression levels of MOG, MBP, CNPase, and PLP in CC were shown by representative blots and statistical graphs (the protein expressions were standardized vs. *β*-actin). Data are reported as means ± SD (*n* = 3/each group); as opposed to the CON group, ^###^*P* < 0.001; as opposed to the CPZ group, ^∗^*P* < 0.05, ^∗∗^*P* < 0.01, and ^∗∗∗^*P* < 0.001.

It is reported that CPZ intake in mice can induce extensive demyelination in many regions of the brain white matter. To evaluate the degree of myelin repair in demyelinated lesions, histological changes in white matter areas, including the lateral corpus callosum (Lat-CC) and medial corpus callosum (Med-CC) [32], anterior commissure (AC), and caudoputamen (CPu) [33] were detected by LFB staining. Compared with the CON group, relative LFB-IOD of Med-CC, Lat-CC, AC, and CPu declined sharply in CPZ mice. Conversely, relative IOD was increased in BSYS-treated mice (Figures 3(a) and 3(b)). Comparable findings were recorded by immunofluorescence staining of MOG and PLP (Figures 3(c) and 3(d)). Compared with vehicle-treated mice, BSYS-treated mice exhibited a stronger recovery from demyelination and a remarkably larger remyelination area 2 weeks after treatment. Moreover, the effects of BSYS in promoting myelin repair were assessed by Western blotting, and the findings illustrated that BSYS treatment dramatically elevated the protein expression levels of CNPase, MBP, MOG, and PLP in the CC comparison with CPZ mice (Figure 3(e)).

### 3.3. BSYS Modulates the Phenotype of Reactive Astrocytes *In Vivo*

**Figure 4:**
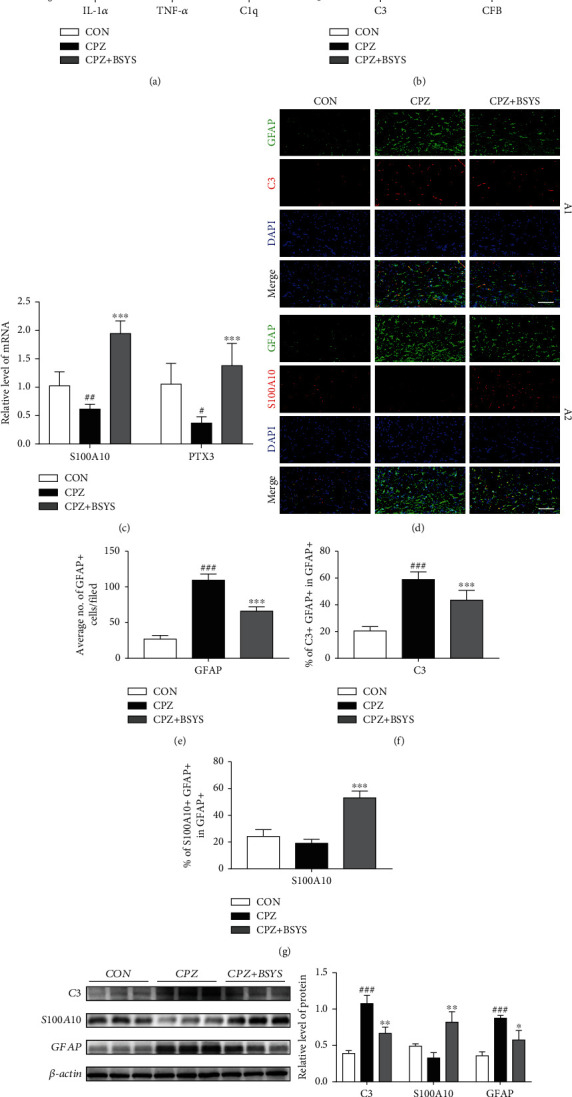
BSYS modulates the phenotype of reactive astrocytes in vivo. (a) Changes in relative expressions of IL-1*α*, TNF-*α*, and C1q in mice per group were determined by qRT-PCR (*n* = 5). (b, c) A1 phenotype markers (CFB, C3) and A2 phenotype markers (PTX3, S100A10) in CC were detected by qRT-PCR (*n* = 5). (d) Immunofluorescence of mice's CC per group utilizing antibodies specific for GFAP (green), C3 (red), and S100A10 (red) (*n* = 3). Scale bars: 100 *μ*m. (e) Measurement of GFAP+ cells density in CC. (f, g) Measurement of the proportion of C3+/GFAP+ and S100A10+/GFAP+ cells in CC. (h) Illustrative Western blotting images and quantitative data of C3, S100A10, and GFAP in CC. Data are displayed as means ± SD; versus the CON group, ^#^*P* < 0.05, ^##^*P* < 0.01, and ^###^*P* < 0.001; in contrast with the CPZ group, ^∗^*P* < 0.05, ^∗∗^*P* < 0.01, and ^∗∗∗^*P* < 0.001.

Previous studies demonstrated that a substantial number of A1 astrocytes were activated in CPZ-induced demyelination lesions [34], and the A1 phenotype was mainly triggered by C1q, TNF-*α*, and IL-1*α* derived from microglia. Thus, the mRNA expression of C1q, TNF-*α*, and IL-1*α* in the brain was quantified by qRT-PCR. The IL-1*α*, TNF-*α*, and C1q mRNA levels in the CC were remarkably upregulated in CPZ mice. However, the mRNA levels were downregulated after BSYS treatment (Figure 4(a)). The gene expression of A1 markers (C3 and CFB) and A2 markers (PTX3 and S100A10) were analyzed to examine the involvement of BSYS in astrocyte polarization. Results illustrated that CPZ markedly enhanced the C3 and CFB expression at week 8 in contrast with the control group. Nevertheless, the administration of BSYS substantially lowered the mRNA levels of A1 markers. When comparing the BSYS-treated group to the CPZ group, levels of A2 astrocyte markers were shown to be elevated in the former (Figures 4(b) and 4(c)).

To confirm whether the results of protein levels were consistent with mRNA levels, immunofluorescence and Western blotting were performed to further identify the astrocytic phenotypic marker. Double-staining was used on brain samples for GFAP+ (astrocytic marker)/C3+ (A1 marker) or GFAP+/S100A10+ (A2 marker). CPZ significantly increased the proportion of A1 astrocytes. However, BSYS administration decreased the numbers of activated astrocytes (GFAP+ cells) and A1 astrocytes (GFAP+/C3+ cells) and raised the ratio of A2 astrocytes in the BSYS groups in contrast with the CPZ group (Figures 4(d)–4(g)). Western blot analysis illustrated that GFAP and C3 were highly expressed, while S100A10 was lowly expressed in the CC of CPZ mice. However, the BSYS administration significantly reversed the trend (Figure 4(h)).

**Figure 5:**
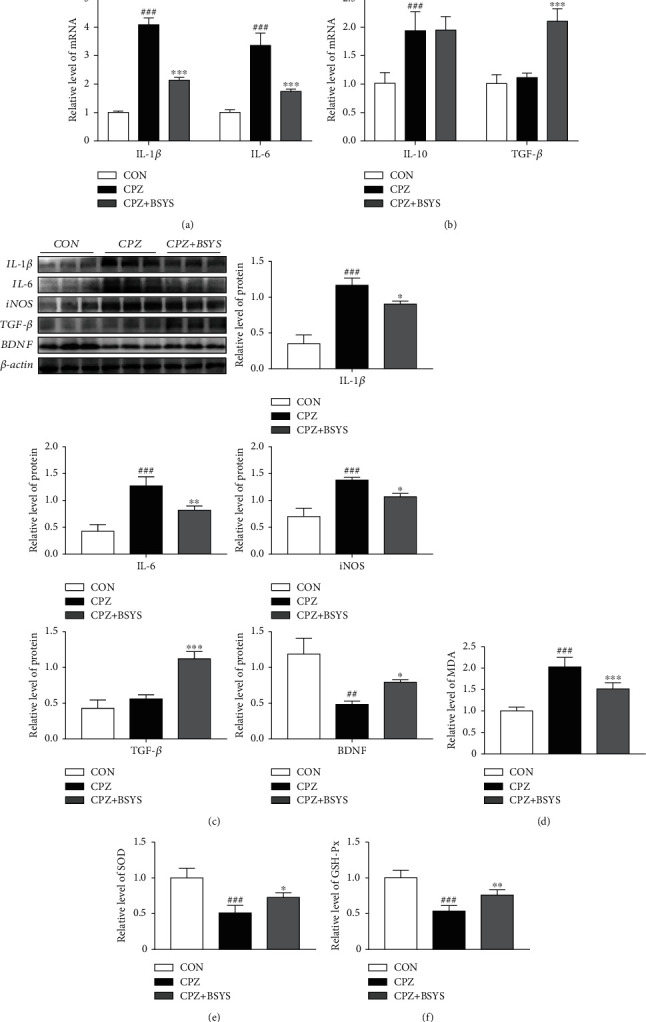
The influence of BSYS on CNS cytokine production and oxidative stress in CPZ mice. (a, b) The levels of anti-inflammatory markers (TGF-*β*, IL-10) and proinflammatory markers (IL-1*β*, IL-6) in CC were identified by qRT-PCR (*n* = 5). (c) Illustrative Western blotting images and quantitative data of proinflammatory cytokines (IL-1*β*, iNOS, and IL-6) and neuroprotective factors (TGF-*β*, BDNF) in CC (*n* = 3). (d) MDA content, (e) SOD levels, and (f) GSH-Px activities of the CC were measured by a corresponding commercial kit (*n* = 5). Data are reported as means ± SD; in contrast with the CON group, ^##^*P* < 0.01, ^###^*P* < 0.001; versus the CPZ group, ^∗^*P* < 0.05, ^∗∗^*P* < 0.01, and ^∗∗∗^*P* < 0.001.

A1 and A2 astrocytes have been confirmed to generate pro- and anti-inflammatory factors, respectively. To further confirm the efficacy of BSYS on the regulation of astrocyte polarization, inflammatory cytokines and neuroprotective factors in CC were detected. qRT-PCR and Western blotting findings illustrated that CPZ markedly increased the expressions of inflammation-associated markers IL-1*β*, IL-6, and IL-10 in contrast with the CON group. BSYS treatment considerably reduced the levels of proinflammatory cytokines IL-6 and IL-1*β* in contrast with the vehicle-treated group. Furthermore, BSYS treatment increased the expressions of TGF-*β* (Figures 5(a) and 5(b)). In addition, Western blotting analysis illustrated that BSYS administration enhanced the expression of BDNF and inhibited oxidative stress factors (inducible nitric oxide synthase (iNOS)) compared to the CPZ group (Figure 5(c)).

Previous studies suggested that CPZ intoxication induces astrocyte activation, while neurotoxic reactive astrocytes increase oxidative stress, which inhibits remyelination [35, 36]. Thus, the impacts of BSYS on the expression of MDA, SOD, and GSH-Px in the CC of CPZ mice were investigated. SOD and GSH-Px levels were dramatically lowered, and the MDA level was markedly elevated in the CPZ group as opposed to the CON group. Interestingly, BSYS administration significantly reduced MDA levels compared with CPZ mice (Figure 5(d)). Treatment with BSYS dramatically enhanced the SOD and GSH-Px expressions in CPZ mice. (Figures 5(e) and 5(f)).

### 3.4. Impacts of BSYS on the miR-155 Signaling Pathway in Demyelinated Mice Brains

**Figure 6:**
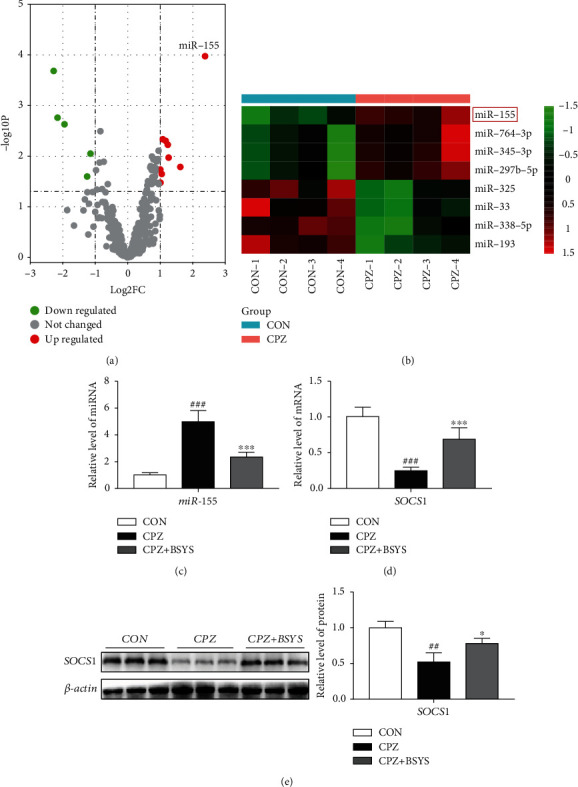
BSYS regulates the miR-155 signaling pathway in the brains of demyelinated mice. (a) Volcano plot and (b) heat map showing the differential miRs between CPZ mice and normal mice. (c, d) The levels of miR-155 and SOCS1 expressions in mice's CC per group as identified by qRT-PCR (*n* = 5). (e) Illustrative blots and statistical graphs of relative protein expression of SOCS1 in CC (*n* = 3). Data are displayed as means ± SD; in contrast with the CON group, ^##^*P* < 0.01, ^###^*P* < 0.001; versus the CPZ group, ^∗^*P* < 0.05, ^∗∗∗^*P* < 0.001.

The microarray data set with the accession number GSE100662 was extracted from the Gene Expression Omnibus (GEO) database to analyze the differences in miRs in CC between CPZ mice and normal mice. The parameters used for screening included |log_2_fold change(FC)| > 1.0 and *P* < 0.05. Utilizing a volcano plot, we identified an aggregate of 14 miRs with differential expression (Figure 6(a)). Under the |*FC*| > 2.0 and *P* < 0.01 parameters, 8 miRNAs with differential expressions are presented in the heat map. CPZ considerably elevated the expression levels of multiple miRs in the CC of mice, especially miR-155 (Figure 6(b)). miR-155 has been proved to inhibit the repair of myelin by promoting a proinflammatory microenvironment in the CNS. Moreover, miR-155 was confirmed to drive the polarization of astrocytes towards a neurotoxic phenotype (A1) via regulating the SOCS1 pathway. Combined with our previous studies, we speculate that BSYS promotes myelin repair by modulating astrocyte polarization via inhibiting miR-155 in a CPZ mouse model.

Next, the effect of BSYS on the miR-155 signaling pathway in CPZ mice was evaluated. qRT-PCR illustrated that the miR-155 expression level in the CC was remarkably elevated in CPZ mice in contrast with normal mice. However, treatment with BSYS significantly decreased relative miR-155 expression in CPZ mice (Figure 6(c)). SOCS1 is a gene that is targeted by miR-155. Several research reports have illustrated that alterations in SOCS1 can modulate the neuroimmune response of astrocytes and might have therapeutic value in MS. Therefore, qRT-PCR and Western blotting were utilized to examine the SOCS1 expression patterns. When CPZ mice were compared with CON mice, the level of SOCS1 expression was found to be lower in the former. However, treatment with BSYS enhanced SOCS1 expression in CPZ mice (Figures 6(d) and 6(e)).

### 3.5. Screening the Optimal Concentration of BSYS-Serum for Astrocytes

**Figure 7:**
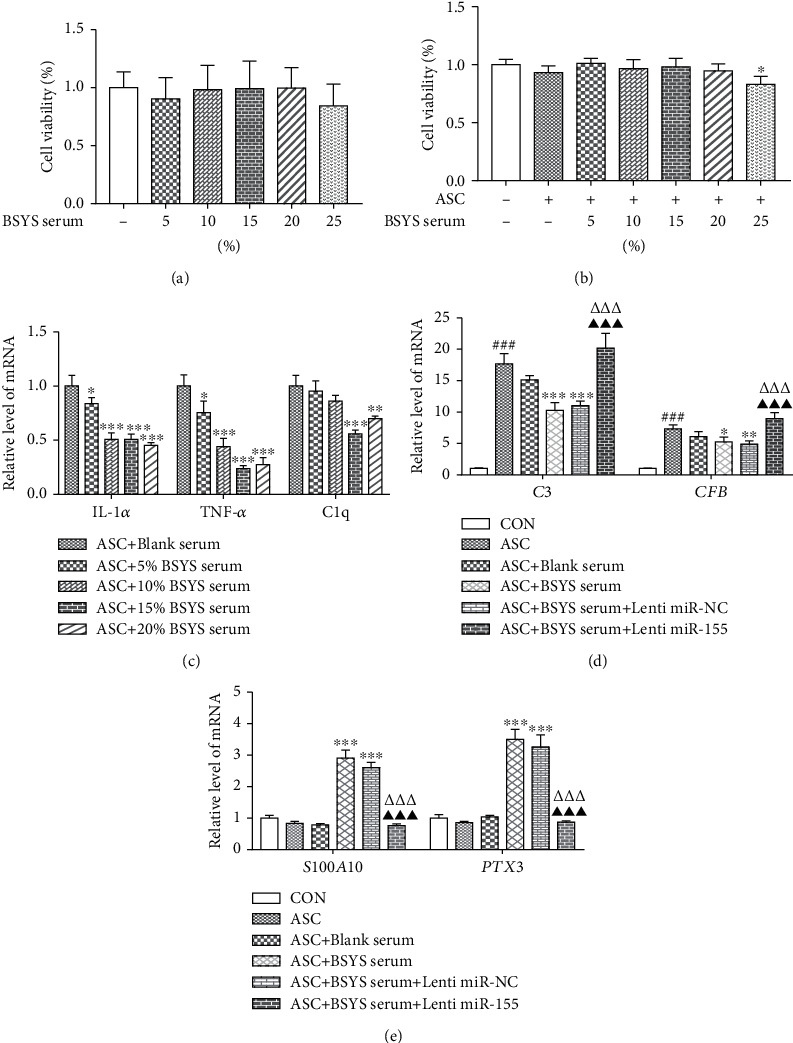
Effect of BSYS-serum on primary astrocytes. (a, b) With or without the stimulation of ASC, astrocytes were treated with 5%-25% BSYS-serum for 24 hours, and the CCK-8 test was utilized to ascertain the viability of the cells. (c) Changes in relative expression levels of C1q, TNF-*α*, and IL-1*α* of astrocytes in each group were evaluated by qRT-PCR. (d, e) A1 phenotype markers (CFB, C3) and A2 phenotype markers (PTX3 and S100A10) in astrocytes were evaluated by qRT-PCR. Data are reported as means ± SD; in contrast to the CON group, ^###^*P* < 0.001; in contrast to the ASC group, ^∗^*P* < 0.05, ^∗∗^*P* < 0.01, and ^∗∗∗^*P* < 0.001; in contrast with the ASC+BSYS group, ^△△△^*P* < 0.001; in contrast with the ASC+BSYS+miR-NC group, ^▲▲▲^*P* < 0.001.

To corroborate the findings of the *in vivo* investigations, BSYS-serum was administrated to primary astrocytes cultured *in vitro*. CCK-8 assay was conducted to ascertain if the concentrations of BSYS-serum used in this experiment influenced the cell viability of astrocytes. CCK-8 assay illustrated that treating astrocytes with 5-25% of BSYS-serum for 24 h had no cytotoxic effect (Figure 7(a)). ASC was used to stimulate astrocytes to investigate the underlying process of A1/A2 astrocyte transition, which is mediated by BSYS. Then, the cell viability (Figure 7(b)) and expression profiles of C1q, TNF-*α*, and IL-1*α* in astrocytes were assessed to determine the optimum concentration of BSYS-serum. C1q, TNF-*α*, and IL-1*α* levels were elevated substantially in ASC-induced astrocytes, and BSYS-serum considerably lowered the expression of these proinflammatory indicators in a dosage-dependent way (Figure 7(c)). The effects of 15% and 20% concentrations were the most significant, but no meaningful difference was discovered between the two concentrations. Therefore, BSYS-serum with a concentration of 15% was selected for subsequent analyses.

### 3.6. BSYS Modulates the Transition of A1/A2 Astrocytes through the Suppression of the miR-155 Expression *In Vitro*

**Figure 8:**
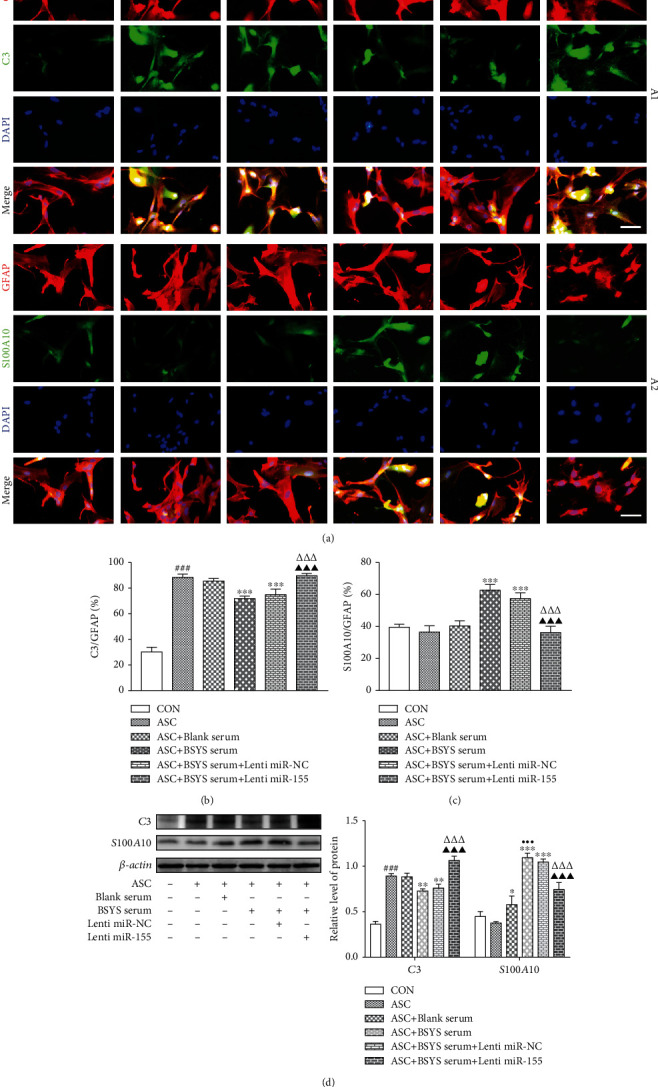
BSYS modulates the A1/A2 Astrocytes transformation by suppressing the miR-155 levels in vitro. (a) Immunocytofluorescence staining of astrocytes per group utilizing antibodies specific for GFAP (red), C3 (green), and S100A10 (green). Scale bars: 50 *μ*m. (b, c) Measurement of the proportion of C3+/GFAP+ and S100A10+/GFAP+ cells in astrocytes. (d) Illustrative Western blotting images and quantitative data of C3 and S100A10 in astrocytes. Data are reported as means ± SD; as opposed to the CON group, ^###^*P* < 0.001; versus the ASC group, ^∗^*P* < 0.05, ^∗∗^*P* < 0.01, and ^∗∗∗^*P* < 0.001; contrasted with the ASC+blank serum group, ^●●●^*P* < 0.001; vs. the ASC+BSYS group, ^△△△^*P* < 0.001; vs. the ASC+BSYS+miR-NC group, ^▲▲▲^*P* < 0.001.

To ascertain if BSYS influenced the level of miR-155, which is required to modulate astrocyte polarization *in vitro*, astrocytes were firstly transfected with miR-155 mimics before ASC stimulation and then treated with BSYS-serum. As displayed in Figure 7(d), the level of the A1 phenotype marker (C3 and CFB) expression was considerably upregulated at 24 h after ASC treatment. This trend was suppressed by BSYS-serum. Nevertheless, the inhibition properties of BSYS-serum on the A1 markers were markedly attenuated after miR-155 mimic administration. Meanwhile, the effect of BSYS-serum promoting the A2 markers (S100A10 and PTX3) was also weakened by transfection with the miR-155 mimics (Figure 7(e)). Immunocytofluorescence staining showed that BSYS-serum lowered the expression level of C3 and elevated the S100A10 expression level in GFAP+ cells in contrast with ASC and blank serum-treated groups, while the effects were considerably diminished in the miR-155 overexpression group (Figures 8(a)–8(c)). Western blot results are consistent with those of immunofluorescence staining (Figure 8(d)).

**Figure 9:**
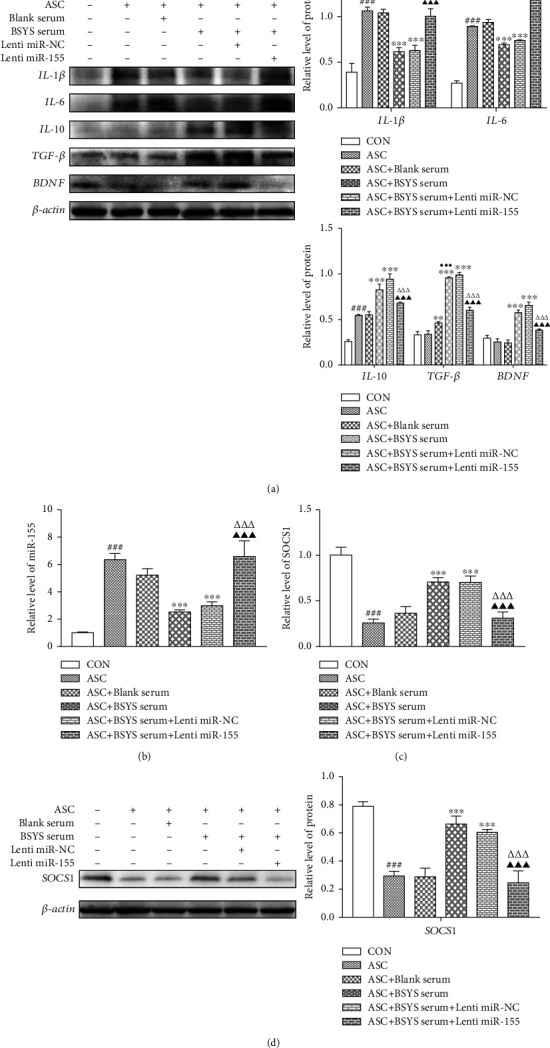
BSYS modulates cytokine levels by regulating miR-155 signaling pathways in astrocytes. (a) Illustrative Western blotting images and quantitative data of inflammatory indicators (IL-6, IL-1*β*) and neuroprotective factors (IL-10, TGF-*β*, and BDNF) in astrocytes. (b, c) The level of miR-155 and SOCS1 expression in astrocytes per group were identified by qRT-PCR. (d) The relative protein expression level of SOCS1 in astrocytes is shown using illustrative blots and statistical graphs. Data are presented as means ± SD; contrasted with the CON group, ^###^*P* < 0.001; contrasted to the ASC group, ^∗∗^*P* < 0.01, ^∗∗∗^*P* < 0.001; contrasted to the ASC+blank serum group, ^●●●^*P* < 0.001; contrasted with the ASC+BSYS group, ^△△^*P* < 0.01, ^△△△^*P* < 0.001; contrasted with the ASC+BSYS+miR-NC group, ^▲▲▲^*P* < 0.001.

As shown in Figure 9(a), levels of neurotoxic markers, such as IL-6 and IL-1*β*, were substantially increased in astrocytes after ASC stimulation but were reversed to the baseline after treatment with BSYS-serum. Furthermore, the low expression of neuroprotective factors in the ASC group, including IL-10, TGF-*β*, and BDNF, were upregulated after BSYS-serum treatment. Similarly, these therapeutic effects were offset by the combination of miR-155-mimic transfection. In addition, miR-155 and its downstream target SOCS1 were detected in astrocytes. It was found that as opposed to the ASC group and the blank-serum treatment group, miR-155 was remarkably inhibited and SOCS1 was upregulated in the BSYS-serum treatment group. However, miR-155 mimic transfection decreased the effects (Figures 9(b)–9(d)). These data suggested that BSYS modulates astrocyte polarization by suppressing the level of miR-155 *in vitro*.

### 3.7. BSYS Stimulates the Oligodendrocytes Maturation In Vitro by Alleviating the Neurotoxic Impacts of A1 Astrocytes

**Figure 10:**
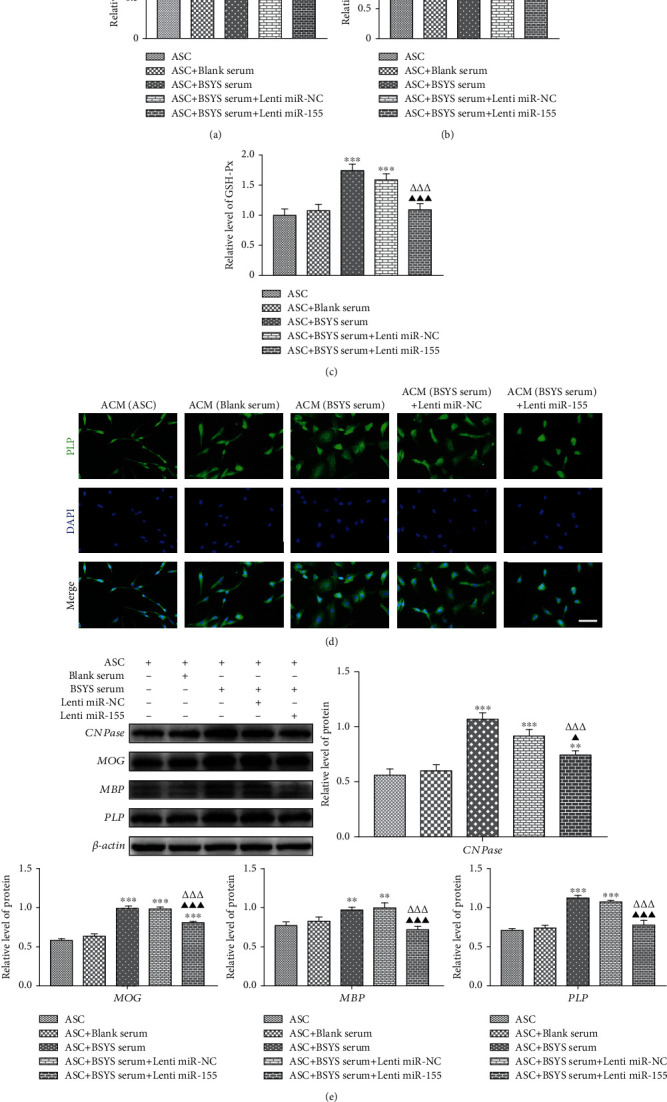
BSYS promotes oligodendrocyte maturation in vitro by ameliorating the neurotoxic impacts of A1 astrocytes. (a) MDA content, (b) SOD level, and (c) GSH-Px activity of OLN-93 cells were determined utilizing the respective commercial kit. (d) The expression of PLP in OLN-93 cells was determined by immunocytofluorescence staining. Scale bars: 50 *μ*m. (e) The relative protein expression level of MOG, PLP, MBP, and CNPase in astrocytes is shown using illustrative blots and statistical graphs. Data are reported as means ± SD; contrasted to the ASC group, ^∗∗^*P* < 0.01, ^∗∗∗^*P* < 0.001; contrasted with the ASC+BSYS group, ^△△△^*P* < 0.001; contrasted to the ASC+BSYS+miR-NC group, ^▲^*P* < 0.05, ^▲▲^*P* < 0.01, and ^▲▲▲^*P* < 0.001.

An earlier study illustrated that A1 astrocytes contribute to oligodendrocytes' death and inhibit OPC differentiation [37] by producing neurotoxic mediators [38]. Neurotoxic reactive astrocytes have been proven to cause oxidative stress, which disrupts oligodendrocyte maturation [39, 40]. Therefore, oxidant biomarkers were measured to evaluate the oxidative stress level in oligodendrocytes [41, 42]. An immature oligodendroglial cell line (OLN-93) was incubated with ACM, and GSH-Px, SOD, and MDA levels were evaluated. Compared with culture medium from A1 astrocytes (A1CM), ACM from BSYS-serum treatment considerably lowered MDA expression level and elevated GSH-Px and SOD levels in OLN-93 cells. Conversely, these therapeutic effects were dramatically attenuated by miR-155 overexpression (Figures 10(a)–10(c)).

Furthermore, to assess the impacts of BSYS on the maturation of oligodendrocytes mediated by A1 astrocytes, PLP expression level in OLN93 cells was detected by immunocytofluorescence. After OLN-93 cells were added to A1CM, low expression of PLP was observed; this phenomenon was counteracted in the BSYS-serum treatment group. However, the effect on promoting oligodendrocyte maturation of ACM in the BSYS-serum-treated group was attenuated by miR-155 transfection (Figure 10(d)). The results of oligodendrocyte maturation markers, including CNPase, MOG, MBP, and PLP in Western blot analysis were consistent with PLP immunofluorescence (Figure 10(e)). It was inferred that BSYS promotes the maturation of oligodendrocytes by alleviating neurotoxic effects of A1 astrocytes via inhibition of miR-155 in vitro.

## 4. Discussion

Multiple sclerosis (MS) is a chronic, disabling neurological condition hallmarked by neurodegeneration and demyelination [43]. In China, the incidence of MS in children is 0.055/100,000 and that in adults is 0.288/100,000 [44]. Numerous studies have been conducted to develop drugs to suppress neuroinflammation and promote myelin repair in patients with MS. Several Chinese herbal medicines have been investigated for their potential to treat MS given their multichannel and multitarget therapeutic effects. BSYS capsule, a traditional Chinese medicine prescription was reported to exert good clinical effects on MS [45]. Preliminary studies from our laboratory showed that BSYS alleviated myelin and axon damage via modulating Th17/Treg [46] and microglia M1/M2 ratio [25] in EAE mice. Additionally, our previous studies also illustrated that treatment with BSYS-serum promoted axonal regeneration *in vitro* [31, 47]. Based on these findings, we examined the mechanisms of the treatment effects of BSYS using the CPZ-mediated demyelination model from the perspectives of antineuroinflammation and remyelination.

Cuprizone is a chelating agent for copper ions which impairs the respiratory chain of oligodendrocytes, leading to oxidative stress and degeneration effects [48]. Continued dietary consumption of CPZ triggers reversible demyelination in distinct brain regions by inducing oligodendrocyte apoptosis [49] and activation of astrocytes and microglia [50]. The CPZ model has been used to explore the mechanisms leading to the destruction and restoration of myelin sheaths, which independently of invading peripheral immune cells [51]. In this research, mice were supplied with a normal diet comprising 0.2% CPZ, which contributed to a remarkable decrease in body weight and impaired motor function. Rotarod behavioral tests showed that BSYS administration ameliorated motor deficits in the CPZ-induced demyelination in mice. CPZ ingestion induced severe demyelination; however, BSYS administration resulted in dense LFB-positive staining in myelin tissues from different brain regions as determined by the LFB staining assay. Moreover, mature oligodendrocytes express myelin proteins such as the MBP and PLP (which are present in compact myelin), CNPase, and MOG, (which are expressed in noncompact myelin) [52, 53]. During the process of remyelination, these proteins agglomerate to neuronal axons and enwrap axons to form myelin sheaths. Consistent with the results of LFB, the expression levels of CNPase, MOG, MBP, and PLP were dramatically elevated in corpus callosum after BSYS treatment, suggesting that BSYS administration during the remyelination phase promoted myelin repair in the CPZ mouse model.

It has been shown that glial cell-mediated neuroinflammation exacerbates oligodendrocyte injury and remyelination failure in the CPZ model [54]. Therefore, modification of the glial cell-mediated neuroinflammatory microenvironment can ameliorate myelin injury and promote repair processes. Myelin fragments stimulate microglia activation in MS [55], which triggers the generation of many cytokines. In particular, TNF-*α*, C1q, and IL-1*α* derived from activated microglia promote inflammation triggered by neurotoxicity astrocytes, referred to as the A1 phenotype [9]. Consistent with the pathological changes of the demyelinated lesions in MS patients, many astrocytes were strongly activated in the demyelinating area of the CPZ mouse model [56]. Several studies have suggested that astrocyte activation leads to a more severe inflammatory response than microglia activation [57]. Evidence from prior studies indicates that the complement component 3 (C3), which is an A1 astrocytes marker, is highly expressed in the demyelinated region. A1 astrocytes inhibit OPC proliferation and differentiation, kill newly generated oligodendrocytes, and promote axon degeneration in demyelinating plaques. On the contrary, the A2 phenotype confers neuroprotective effects, thereby increasing the number of mature oligodendrocytes and inhibiting white matter injury [58]. It has been discovered that A2 astrocytes specifically express the proteins S100A10, PTX3, and TGM1 [59]. As a corollary, regulation of the conversion of reactive astrocytes from A1 to A2 will inhibit neuroinflammation and promote myelin repair, thus accelerating MS recovery. In light of these observations, we investigated whether astrocyte polarization mediated the effects of BSYS on the CPZ mouse model. Results showed that a substantial number of A1 astrocytes were found in the lesions caused by demyelination. Interestingly, BSYS treatment significantly prevented A1 formation and increased A2 astrocytic polarization in the demyelination area.

Astrocytes regulate CNS immune responses and neuroinflammation during MS [60]. A1 astrocytes are known to be critical sources of proinflammatory and oxidative stress markers, including iNOS, IL-6, TNF-*α*, and IL-1*β*, and can cause neurotoxicity [61]. The demyelination and remyelination processes in MS lesions are influenced by various proinflammatory chemokines and cytokines. Astrocyte-derived IL-1*β* and TNF-*α* have been reported to strongly inhibit OPC differentiation and oligodendrocyte survival [62, 63]. Therefore, reducing the production of proinflammatory factors by restraining A1 polarization may promote oligodendrocyte growth and myelin repair. In this study, *in vitro* experiments revealed that A1 astrocytes increased C3 and CFB mRNA levels and also the staining intensity and protein expression of C3. However, treatment with BSYS drug-containing serum after ASC stimulation decreased the A1 phenotype and lowered the IL-6 and IL-1*β* levels. These results were in agreement with those obtained from *in vivo* studies. Interestingly, the expression levels of A2 astrocyte markers, S100A10 and PTX3, were increased after BSYS administration *in vivo* and *in vitro*. BSYS treatment increased the secretion of neuroprotective and anti-inflammatory markers, including BDNF, TGF-*β*, and IL-10, from astrocytes to enhance the capacity of oligodendrocytes to differentiate and mature, thus facilitating the remyelination process.

Moreover, neurotoxic reactive astrocytes have been demonstrated to cause oxidative stress, which hinders the differentiation and maturation of oligodendrocytes. The prooxidant biomarker, MDA, serves as the end product of lipid peroxidation. The SOD and GSH-Px can remove free radicals and are regarded as crucial antioxidative enzymes. A1 astrocytes culture supernatants cocultured with OLN-93 cells resulted in the abnormal expression of MDA, SOD, and GSH-Px. However, this phenomenon was reversed by BSYS-serum treatment. Moreover, the culture supernatants from astrocytes treated with BSYS-serum significantly increased the expression levels of oligodendrocyte maturation-related proteins, including CNPase, MOG, MBP, and PLP. These findings suggest that BSYS exerts proremyelination effects by improving oligodendrocyte differentiation and maturation, a process mediated by astrocyte polarization. However, the molecular mechanism by which BSYS induces astrocyte polarization remains uncertain.

Although the mechanism underlying A1/A2 phenotype transformation by astrocytes is not well understood, several studies have shown that some miRs, especially miR-155, modulate astrocytic polarization. It has been demonstrated that miR-155 overexpression in activated astrocytes drives astrocytic polarization towards the A1 proinflammatory phenotype. By contrast, miR-155 downregulation in astrocytes decreases the generation of proinflammatory factors and astrocyte-mediated neurotoxicity via enhancing the expression of suppressor of cytokine signaling 1 (SOCS1) [17]. SOCS1 is an intracellular, cytokine-inducible protein that restrains cytokine signaling in astrocytes and other inflammatory immune cells in the CNS, thereby alleviating neuroinflammation [64]. It has been demonstrated to play therapeutic roles against MS [65]. In previous studies, the expression of miR-155 was considerably upregulated in reactive astrocytes [66] and white matter lesions of MS tissue samples [67, 68]. Similarly, a substantial elevation of the miR-155 expression level has been reported in brain samples of the CPZ-mediated demyelination mouse model. These findings illustrate that the miR-155/SOCS1 axis participates in myelin damage and astrocyte polarization. Hence, the miR-155 pathway is a viable treatment target for MS. Indeed, we previously found that BSYS regulated miR-155 and miR-124 to facilitate microglial M2 polarization and reduce demyelination in EAE mice. However, the mechanisms through which BSYS regulates astrocyte polarization-mediated remyelination are unclear. In this study, the regulatory function of BSYS on A1/A2 phenotype transformation and promoting effect on oligodendrocyte maturation was significantly attenuated following miR-155 overexpression in astrocytes *in vitro*. In the future, we will combine gene knockout and knockin techniques to further clarify the effect of BSYS on the link between miR-155 and astrocytic polarization in a mouse model of MS.

## 5. Conclusions

Collectively, the results reported here demonstrate that BSYS ameliorates neuroinflammatory and promotes myelin repair by modulating astrocytic polarization and secretion of pro-/anti-inflammatory factors. Mechanistically, we show that BSYS regulates A1/A2 polarization by inhibiting miR-155 leading to the upregulation of SOCS1 signaling pathways. Therefore, BSYS might present a promising therapeutic agent for the management of MS as well as other CNS demyelinating disorders.

## Data Availability

The data that support the findings of this study are available from the corresponding author upon reasonable request.

## Supplementary Materials

Supplementary Materials Supplementary Table 1: primer sequences.
