# Supplementary material for: Bu Shen Yi Sui Capsule Promotes Myelin Repair by Modulating the Transformation of A1/A2 Reactive Astrocytes In Vivo and In Vitro
Source: Oxid Med Cell Longev. 2022 Sep 1;2022:3800004. doi: 10.1155/2022/3800004 (PMC9458373; doi:10.1155/2022/3800004)
Supplement: Supplementary Materials — Supplementary Table 1: primer sequences. [file 3800004.f1.docx]

| Genes | Primer sequence, 5'-3' | |
| --- | --- | --- |
|  | Forward | Reverse |
| IL-1α | CGCTTGAGTCGGCAAAGAAAT | CTTCCCGTTGCTTGACGTTG |
| C1q | TCTGCACTGTACCCGGCTA | CCCTGGTAAATGTGACCCTTTT |
| TNF-α | TGTGCTCAGAGCTTTCAACAA | CTTGATGGTGGTGCATGAGA |
| C3 | GCAGACCTTAGCGACCAAGT | CCGCAATGACTGTTGGTGTC |
| CFB | GGATGTCAAAGCTCTGTTTGTATC | CTTTCTTGTCCCCATTCTTGATGTA |
| S100A10 | CCTCTGGCTGTGGACAAAAT | CTGCTCACAAGAAGCAGTGG |
| PTX3 | AACAAGCTCTGTTGCCCATT | TCCCAAATGGAACATTGGAT |
| IL-1β | TCCAGGATGAGGACATGAGCAC | GAACGTCACACACCAGCAGGTTA |
| IL-6 | TCTGCAAGAGACTTCCATCCAGT | GTGAAGTAGGGAAGGCCGTG |
| IL-10 | GCGGCTGAGGCGCTGTCAT | GGCCTTGTAGACACCTTGGTCTTGG |
| TGF-β | GGACTCTCCACCTGCAAGAC | GACTGGCGAGCCTTAGTTTG |
| SOCS1 | CACCTTCTTGGTGCGCG | AAGCCATCTTCACGCTGAGC |
| β-actin | ATATCGCTGCGCTGGTCGTC | AGGATGGCGTGAGGGAGAGC |

Supplementary Table 1: Primer sequences
